# COVID‐19‐associated pseudothrombocytopenia

**DOI:** 10.1002/jha2.239

**Published:** 2021-06-06

**Authors:** Ruben Van Dijck, Mandy N. Lauw, Maurice Swinkels, Henk Russcher, A.J. Gerard Jansen

**Affiliations:** ^1^ Department of Haematology Erasmus MC University Medical Center Rotterdam Rotterdam The Netherlands; ^2^ Department of Clinical Chemistry Erasmus MC University Medical Center Rotterdam Rotterdam The Netherlands

Coronavirus disease 19 (COVID‐19), caused by infection with severe acute respiratory syndrome coronavirus 2 (SARS‐CoV‐2), can be associated with changes in platelet count [1, 2]. Thrombocytopenia has been reported in up to 40% of COVID‐19 infections [3, 4, 5] and is an important marker for morbidity and mortality [1, 2, 5]. Hence, monitoring of platelet counts is important in diagnosis and treatment of COVID‐19 patients. Thrombocytopenia can be a result of the COVID‐19 infection itself (septicaemia), diffuse intravascular coagulation (DIC), medication or a COVID‐19‐associated immune thrombocytopenic purpura (ITP) [6]. A rare and often missed alternative explanation of thrombocytopenia is pseudothrombocytopenia [7]. Pseudothrombocytopenia or spurious thrombocytopenia is an in vitro phenomenon of platelet agglutination caused by an anticoagulant, usually ethylenediaminetetraacetic acid (EDTA), resulting in a falsely lowered automated platelet count [8]. The mechanism of pseudothrombocytopenia is not clearly defined, but it is suggested to be an immunologically mediated phenomenon of platelet clumping due to the formation of immune complexes between naturally occurring autoantibodies and cryptic epitopes of the glycoprotein IIb/IIIa complex on the platelet membrane that are exposed by the EDTA anticoagulant used for routine blood sample collections [9]. This phenomenon has been previously reported to be associated with autoimmune diseases and infections [10], such as hepatitis A [11], mononucleosis [12] and *Plasmodium falciparum* malaria [13]. It has a reported incidence between 0.03% and 0.27% among the general population [14]. Here, we report the first patient with pseudothrombocytopenia related to COVID‐19 infection and its natural course.

Our patient is a 54‐year‐old woman with a history of sarcoidosis diagnosed in October 2019, for which she was still being treated with daily prednisolone 7.5 mg (December 2019) and weekly methotrexate 12.5 mg (January 2020). She previously had a stable normocytic anaemia and normal platelet counts. Approximately 10 days after onset of respiratory symptoms, she presented with progressive respiratory failure (and a need for high‐flow oxygen therapy) secondary to COVID‐19 bilateral pneumonia with a positive SARS‐CoV‐2 polymerase chain reaction (PCR) test on day of presentation in our hospital. After a chest CT‐angiography scan excluded pulmonary embolism, empirical treatment according to local protocol with dexamethasone (prednisolone was discontinued on admission), ceftriaxone, ciprofloxacin and prophylactic low‐molecular weight heparin (nadroparin) was started. A full blood count on the day of presentation showed a stable haemoglobin level of 9.9 g/dl, with normal leukocyte (7.0 × 10^9^/L) and platelet (236 × 10^9^/L) counts. The following day, a marked fall in platelet count to 54 × 10^9^/L was noted (measured with the Coulter impedance method on EDTA)—a trend that continued the following days to a nadir platelet count of 6 × 10^9^/L on day 10 after presentation (Figure 1). On this day, dexamethasone and methotrexate were discontinued; the latter because a possible causal relationship with the thrombocytopenia was postulated. Further laboratory work showed a slightly elevated lactate dehydrogenase (427 U/L), normal haptoglobin, normal prothrombin (PT) and activated partial thromboplastin time (aPTT) and elevated D‐dimers (3.50 mg/L), excluding a thrombotic microangiopathy. No further testing for diffuse intravascular coagulation (DIC) or heparin‐induced thrombocytopenia (HIT) was performed at this time. HIV‐serology test was negative. There were no clinical signs of bleeding or thrombosis. On day 10 after presentation, platelet count in a citrate blood sample was 129 × 10^9^/L. Analysis of a peripheral blood film at the same moment showed platelet agglutination in EDTA as well as in citrate, although much less evident (Figure 2). Hence, the diagnosis of pseudothrombocytopenia was confirmed, which also marked the misdiagnosis of a true thrombocytopenia at first in this patient.

**FIGURE 1:**
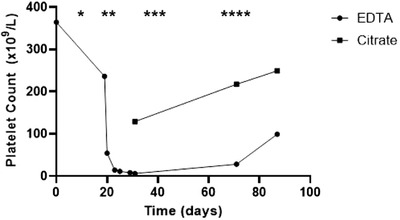
Platelet counts in EDTA and citrate anticoagulated blood in our COVID‐19 infected patient with pseudothrombocytopenia. (*) Onset of respiratory symptoms. (**) Start of hospitalization and positive SARS‐CoV‐2 PCR test. (***) Discharge from hospital. (****) Positive SARS‐CoV‐2 IgM and total antibody ELISA (CE‐IVD) indicating SARS‐CoV‐2 seroconversion. Starting from the moment of hospitalization, our patient received treatment with ceftriaxone and ciprofloxacin for 5 consecutive days, dexamethasone for 10 consecutive days and prophylactic nadroparin until discharge

**FIGURE 2:**
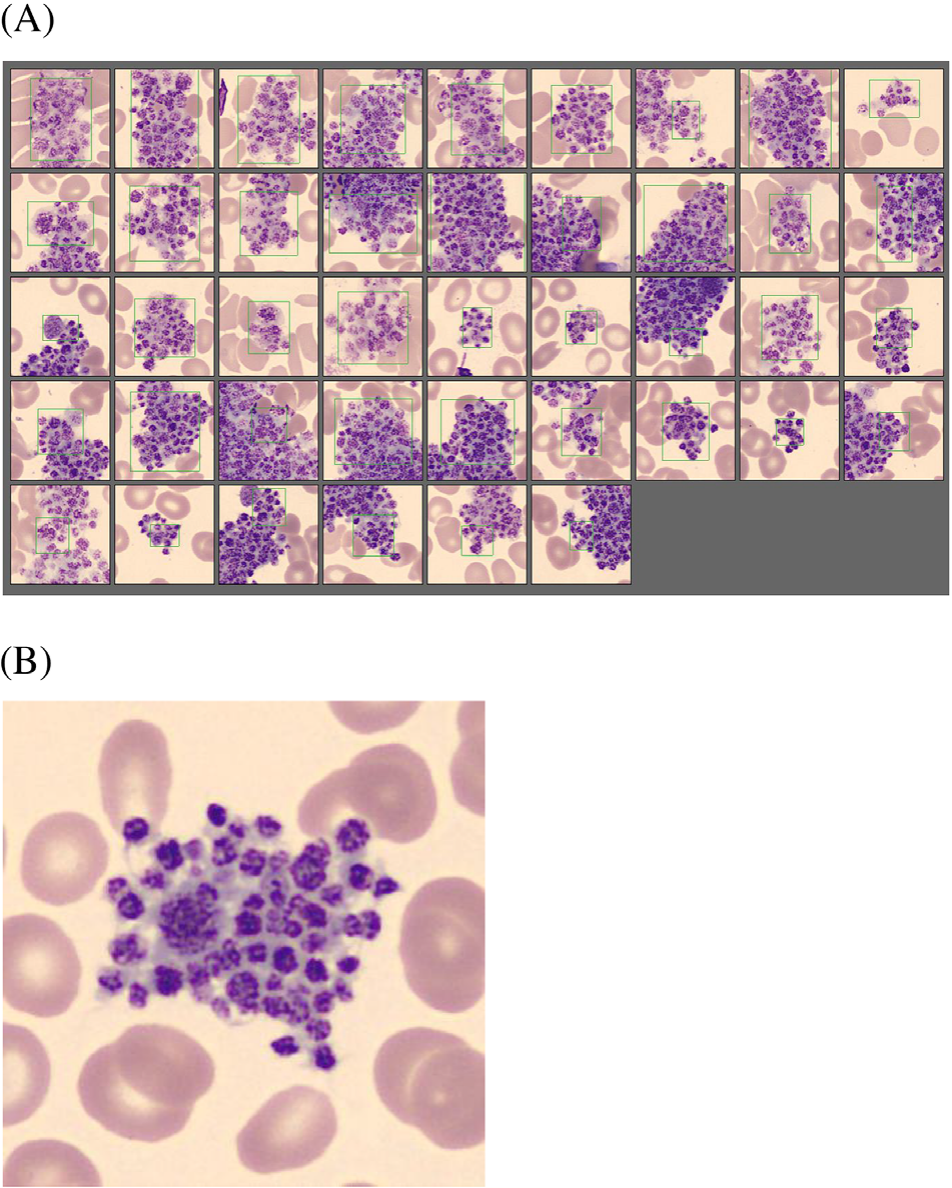
Peripheral blood smear showing platelet agglutination in EDTA in our COVID‐19 infected patient with pseudothrombocytopenia. (A) Picture made with CellaVision (DM96) microscope. Original magnification ×100 for each panel. All inserts refer to the same blood sample. This blood smear was performed on the day of the nadir platelet count, which was 10 days after diagnosis of COVID‐19 infection, and very convincingly shows platelet agglutination in EDTA anticoagulated blood, indicating pseudothrombocytopenia. (B) Picture made with CellaVision (DM96) microscope. Original magnification ×100. Detail of the same blood smear showing platelet agglutination in EDTA anticoagulated blood

In the following weeks, together with SARS‐CoV‐2 seroconversion and clinical recovery, we noted a positive trend in platelet counts (Figure 1): EDTA 28 × 10^9^/L and citrate 217 × 10^9^/L in week 6 after nadir, EDTA 99 × 10^9^/L and citrate 249 × 10^9^/L in week 8 after nadir. The phenomenon seems transient, as it was reported to be in the only other publication describing a similar case, although platelet transfusion was given in this case [7]. SARS‐CoV‐2 IgM and total antibodies were first measured, using the Wantai ELISA‐test (WS‐1196 and WS‐1096), in week 7 after diagnosis of COVID‐19 infection, already showing sufficient SARS‐CoV‐2 seroconversion. In COVID‐19‐related pseudothrombocytopenia, we suggest a possible link with SARS‐CoV‐2 IgM antibodies and hypothesize an EDTA‐dependent immune‐complex formation with cryptic platelet membrane epitopes. To test this, we incubated patient serum with EDTA‐blood of a universal donor, but this did not induce platelet agglutination. Taken together, this may suggest that generation of cryptic epitopes is patient specific.

In conclusion, we illustrate the importance of considering pseudothrombocytopenia in COVID‐19‐associated thrombocytopenia. This is the first case of COVID‐19‐associated pseudothrombocytopenia in which we also describe the transience of this diagnosis. It is essential to recognize this in vitro phenomenon, as this falsely lowered automated platelet count is not associated with a clinical bleeding tendency, does not have any therapeutic consequences (platelet transfusion nor discontinuation of essential medication) and is self‐limiting, as shown in our patient. In the differential diagnosis of COVID‐19‐associated thrombocytopenia, exclusion of pseudothrombocytopenia is therefore critical.

## CONFLICT OF INTEREST

The authors declare that there is no conflict of interest.

## AUTHOR CONTRIBUTIONS

R. Van Dijck, M.N. Lauw and A.J.G. Jansen conceived the idea. R. Van Dijck wrote the manuscript. R. Van Dijck and H. Russcher provided Figures 1 and 2. A.J.G. Jansen, M.N. Lauw, M. Swinkels and H. Russcher reviewed and critically evaluated the manuscript. All authors approved the final version of the manuscript.
